# Supplementary material for: Community-based model of care to improve access to and utilization of health services for common non-communicable diseases among culturally and linguistically diverse populations in Australia: an implementation research protocol
Source: Arch Public Health. 2026 Jun 10;84:186. doi: 10.1186/s13690-026-01986-0 (PMC13479535; doi:10.1186/s13690-026-01986-0)
Supplement: Supplementary file 1 [file 13690_2026_1986_MOESM1_ESM.docx]

**Supplementary File 1. Expanded Operational Procedures and Prespecified Statistical Analysis Plan for An Implementation Research Protocol**

**Title of main protocol:** Community-based model of care to improve access to and utilization of health services for common non-communicable diseases among culturally and linguistically diverse populations in Australia: An implementation research protocol

**Purpose**: This supplementary file is prepared to provide detailed operational procedures, clearly defined outcome measures, and a prespecified statistical analysis plan, all defined a priori and aligned with the phases outlined in the main protocol. It is integral component of the study protocol and should be read alongside the main manuscript. The study sequence is as follows: Scoping Review → Quantitative Survey → Qualitative Interviews → Model Co-development → Implementation → Outcome Assessment.

# Section 1. Formative Phase: Scoping Review, Quantitative Survey, Qualitative Interviews

## 1.1 Scoping Review Methods

The scoping review will be conducted in accordance with the Preferred Reporting Items for Systematic Reviews and Meta-Analyses extension for Scoping Reviews (PRISMA-ScR) guideline.

**Objective**: Identify attributes of successful community-based non-communicable disease (NCD) care models that enhance health care access and utilization for hypertension and/or diabetes screening services among culturally and linguistically diverse (CALD) populations, and map relevant barriers and /or facilitators.

**Review question:** What models, implementation strategies, and contextual determinants have improved access and utilization of screening services for hypertension and diabetes in CALD or analogous populations, and what are their reported outcomes?

**Eligibility criteria (PCC):** Population-CALD or culturally diverse/migrant community adult members; Concept-community-based models to improve NCD health care access/utilization (hypertension, diabetes); Context-healthcare/community settings internationally, with emphasis on Australia.

**Sources**: Databases-PubMed/MEDLINE, Embase, Scopus, Web of Science; Grey literature-government and organizational reports; hand-search of reference lists.

**Search limits:** English; year range: 2000 to present (to capture modern community-based NCD interventions).

**Study selection:** Two reviewers will independently screen titles/abstracts, then full texts; disagreements will be resolved by consensus or a third reviewer. Selection process will be documented in the PRISMA-ScR flow diagram.

**Data charting:** Author/year, country, population (CALD descriptors), setting, intervention components, implementation strategies, comparators, outcomes, implementation determinants (barriers/facilitators).

**Synthesis:** Descriptive mapping of intervention components and contexts will be undertaken, followed by a narrative synthesis organized by Reach, Effectiveness, Adoption, Implementation, and Maintenance (RE-AIM) and/or Consolidated Framework for Implementation Research (CFIR) constructs. Components for model co-development workshops will be identified based on the scoping review and used exclusively to guide the development of interventions.

## 1.2 Quantitative Survey Methods

**Objective**: Characterize CALD communities’ service preferences, barriers/facilitators, and baseline screening/utilization patterns for hypertension and diabetes and inform model co-development process.

**Instrument development:** Survey built in Qualtrics; domains include sociodemographics; healthcare access and navigation; prior screening/testing; knowledge of hypertension/diabetes; perceived barriers (language, transport, cost, appointment availability, cultural concordance); service delivery preferences (location, timing, interpreter needs); trust and satisfaction; digital access.

**Content validity:** Expert review and patient and public involvement (PPI) input and consecutive pilot testing with 20 participants to refine wording and completion time.

**Sampling frame and size:** CALD adults ≥40 years residing in Brisbane City; minimum n = 384 (95% CI, 5% margin of error). Recruitment via Qualtrics existing Pannel.

**Data collection:** Data will be collected through Qualtrics following the provision of signed informed consent, and participant anonymity will be maintained. The data will be collected only once during the formative phase prior to model co-development.

**Planned analyses:** Descriptives with 95% CIs; comparisons by sex, age and possibly continent, and region or language groups (χ², t/Wilcoxon if appropriate). In addition, regression analysis for explanatory factors will be considered. The survey findings will be used to characterize baseline access patterns and service preferences and to inform model co-development.

**Note**: The Qualtrics survey is formative only and will not be used for the baseline DiD survey. The DiD uses site‑specific registry/logs at pre‑ and post‑implementation periods.

## 1.3 Qualitative Interviews Methods

**Objective:** Explore lived experiences, preferences, and determinants of health care access/utilization among CALD adults, and implementation determinants among providers.

**Sampling:** Purposive maximum-variation sampling across ethnicity, language, age, gender, migration pathways, and healthcare experience; target n = 12-15 CALD service users and n = 12-15 health care providers, with final numbers to be determined by information saturation.

**Data collection:** Semi-structured interview guides (CALD community and health care provider versions). Interviews will be conducted in participant’s preferred language with certified interpreters as needed; audio-recorded with consent; field notes on context and nonverbal cues. The interviews will be conducted once during the formative phase and will focus on contextual determinants and experiential insights not captured through the quantitative survey.

**Analysis:** Data will be analyzed using reflexive thematic analysis with a hybrid inductive-deductive coding approach, mapped to CFIR and/ or RE-AIM constructs. Reflexive memos will be maintained, and analytic discussions with PPI contributors will support credibility.

**Trustworthiness**: Analyst triangulation, transparent audit trail in NVivo, purposive sampling for breadth, member-checking.

# Section 2. Model Co-Development Phase

**Structure**: Three iterative workshops: (1) evidence and formative findings presentation + prioritization; (2) design of model components; (3) refinement and validation.

**Decision process:** Nominal Group Technique with consensus ≥70% to include components; disagreements deferred to advisory group for adjudication.

**Standardisation categories:** (a) Mandatory, (b) Optional, (c) Prohibited; Adaptation levels: (i) Exactly, (ii) With boundaries, (iii) Flexible. Examples: Mandatory/Exactly - standardized BP measurement checklist; With boundaries, 2-4 week recheck window; Flexible-education modality (group vs. one-on-one vs. digital). All adaptations to intervention components will be prospectively documented and reviewed to ensure fidelity to core functions while allowing contextual tailoring, with major adaptations reviewed by the project advisory group.

**Logic model:** Inputs → Activities → Outputs → Outcomes (screening uptake, linkage to care, control etc.) → Impact (reduced inequities in hypertension outcomes).

# Section 3. Implementation Phase

**Sites**: Six interventions and six matched control faith-based organizations will be included. Potential matching criteria include CALD population size, demographic composition (age, sex, region of birth) if feasible. Clear documentation of matching procedures will be maintained for transparency and reproducibility.

**Training**: Project facilitators will complete a structured curriculum comprising 4-5 interactive sessions covering: (1) cultural safety and community engagement principles, (2) hypertension and diabetes screening protocols, (3) referral pathways, (4) documentation and data security, and (5) fidelity monitoring procedures. Pre- and post-training knowledge assessments will be conducted, with an ≥80% threshold for competency. Facilitators scoring below 80% will undergo targeted retraining.

**Coordinators and facilitators**: Each site will have one designated facilitator responsible for overseeing implementation fidelity, ensuring accurate and timely data capture, providing on-site coaching, and escalating issues to the project-level coordinator. A central project coordinator will provide overarching supervision, troubleshooting, and consistency across sites.

**Workflow**: Community registry → invitation → consent → counseling and screening (BP and HbA1c/random glucose) → result notification and counseling → referral for confirmation/treatment as required → follow-up tracking of outcomes.

**Fidelity monitoring:** Fidelity will be assessed quarterly using a standardized checklist capturing adherence to core components, intervention dose delivered, quality of delivery, and participant responsiveness. Corrective actions may include on-site coaching or targeted retraining. Persistent noncompliance will be escalated to the project advisory group for resolution. All fidelity assessments and corrective actions will be documented systematically.

**Risk management:** All study procedures are classified as low risk. An adverse event log will be used to capture any unforeseen events, with each incident reviewed within 72 hours by the project coordinator. Escalation procedures and mitigation strategies will be documented for audit purposes.

# Section 4. Outcome Measures (Operational Definitions)

The primary effectiveness outcome is “Reach”, defined as the site‑period proportion of eligible CALD adults who complete hypertension and/or diabetes screening during each measurement period (baseline and follow‑up). The primary implementation outcomes are acceptability, appropriateness, and feasibility of the model of care, measured at the site and stakeholder level. Secondary outcomes include follow-up completion, treatment initiation, clinical control (BP and glycemia), intervention fidelity, program cost, and sustainability. Baseline measurements will be collected prior to implementation, with follow-up assessments occurring at 6-12 months post-implementation depending on the specific outcome.

**Note**: Individuals who screen positive during each period will form a **nested cohort** and followed longitudinally for their respective clinical pathway outcomes (e.g., confirmatory diagnosis within 4-8 weeks; treatment initiation within 4 weeks; BP/glycaemic control at clinical follow‑up). All clinical pathway outcomes will be aggregated and analysed **as site‑period proportions**, maintaining consistency with the repeated cross‑sectional design.

**Table 1.** Implementation outcomes under RE-AIM plus clinical process outcomes; time points at baseline and 6-12 months post-implementation.

| Outcome | Operational definition | Numerator / Denominator | Data source & timing | Notes / Instruments |
| --- | --- | --- | --- | --- |
| Reach | Proportion of invited eligible CALD adults screened for BP and diabetes | No. screened / No. invited | Community registry & screening logs; baseline and follow-up | RE-AIM construct |
| Follow-up completion rate | Screen-positive individuals completing confirmatory diagnosis and/or treatment initiation within 4-8 weeks | No. completed follow-up / No. screen-positive | Clinical records; 4-8 weeks from screen | Process effectiveness |
| Treatment initiation | Newly diagnosed individuals started on recommended therapy | No. initiated / No. newly diagnosed | Clinical records; within 4 weeks of confirmation | Process effectiveness |
| BP control | Among diagnosed hypertension: <140/90 mmHg at follow-up | No. controlled / No. with hypertension | Clinic measurements; 6 months | Clinical outcome |
| Glycemic control | Among diagnosed diabetes: HbA1c <7.0% at follow-up | No. controlled / No. with diabetes | Pathology/clinic records; 6 months | Clinical outcome |
| Adoption | Staff delivering ≥80% of assigned model components | No. staff meeting threshold / No. staff trained | Training & supervision logs; quarterly | Site-level implementation |
| Acceptability | Stakeholder perception of model satisfaction/agreement | Mean AIM score | AIM survey; post-implementation | AIM (4 items) |
| Appropriateness | Perceived fit/relevance of model to context | Mean IAM score | IAM survey; post-implementation | IAM (4 items) |
| Feasibility | Perceived practicality of implementation | Mean FIM score | FIM survey; post-implementation | FIM (4 items) |
| Fidelity | Adherence to core components and quality of delivery | Fidelity score / 100% | Checklist; quarterly | Adherence, dose, quality, responsiveness |
| Cost | Program costs from provider perspective | Total & unit costs; ICER per additional person screened | Costing tool & TDABC logs; ongoing | Time-driven activity-based costing |
| Sustainability | Capacity to maintain program domains over time | CSAT/PSAT composite scores | CSAT/PSAT; endline and 6–12 months later | Sustainment (EPIS/RE-AIM) |

Clinical measurement protocols: BP-seated, arm supported, avoid caffeine/smoking 30 min; 3 readings, average last two. Diabetes-HbA1c preerred; random glucose if HbA1c infeasible; confirmation via standard clinical pathways.

# Section 5. Prespecified Statistical Analysis Plan (Before-After DiD)

## 5.1 Objectives and Hypotheses

**Primary outcome:** The primary outcome for the before-after DiD analysis is Reach, defined as the site‑period proportion of eligible CALD adults completing hypertension and/or diabetes screening at baseline and again at 6-12 months post‑implementation. Reach is a site‑period measure and will not require tracking the same individuals across time.

**Primary objective:** To estimate the causal effect of the community‑based model of care on Reach using a before-after DiD design across intervention and matched control sites.

**Primary hypothesis**: Compared with control sites, intervention sites will experience at least a 20 percentage-point increase in Reach from baseline to follow-up.

## 5.2 Populations

Primary analysis includes all site-period observations (baseline and follow-up) from the six intervention and six matched control clusters. Individual‑level samples drawn within each period will be used to describe covariates, and when pooled across sites, analyses will apply sampling/design weights to address unequal inclusion probabilities. Individuals who screen positive will form a **nested cohort** and will be used only for secondary analyses involving follow‑up completion, treatment initiation, and clinical control outcomes. These nested‑cohort outcomes will be aggregated and analyzed as site‑period proportions, consistent with the repeated cross‑sectional framework.

## 5.3 Sample Size and Power

The planned total sample size for the study is approximately 1,200 participants, with around 100 participants recruited from each cluster across the six intervention and six control sites. The sample size calculation assumes a baseline screening rate of 50% in both groups and a post‑intervention increase to 70% in the intervention clusters, with no change expected in the control clusters. To account for the clustered study design, a design effect (DE) was applied using the formula DE = 1 + (m − 1) × intra-cluster correlation coefficient (ICC). Based on an average cluster size (m) of 100 participants and an assumed ICC of 0.02, the corrected design effect is calculated as DE = 1 + (100 − 1) × 0.02 = 2.98. This adjustment increases the required sample size relative to an unadjusted individual-level calculation. The planned total sample size of 1,200 participants therefore exceeds the minimum required to detect the hypothesized 20‑percentage‑point increase in screening uptake with 80% power and a two‑sided α of 0.05.

## 5.4 Primary Impact Model (Before-After DiD)

A before-after DiD model will be used to estimate the intervention effect on Reach. Reach will be modelled as a site‑period outcome, consistent with the repeated cross‑sectional design. For individual‑level exploratory models, screening completion (Y_ist_) will be represented as:

$$Y_{ist}=\beta_{0}+\beta_{1}\text{Post}_{t}+\beta_{2}\text{Intervention}_{s}+\beta_{3}(\text{Post}_{t}\times\text{Intervention}_{s})+\gamma X_{ist}+\varepsilon_{ist}$$

**Where:**

- $Y_{ist}$= binary indicator of hypertension/diabetes screening completion
- $\text{Post}_{t}$= indicator for post-implementation period
- $\text{Intervention}_{s}$= indicator for intervention site
- $X_{ist}$= vector of covariates (age, sex, region of birth, education, primary language, baseline comorbidities)
- $\beta_{3}$= DiD estimate (effect of the intervention)
- $\varepsilon_{ist}$= individual-level residual error

**Estimation**: The primary analysis method will be a Generalized Estimating Equations (GEE) framework with either a logit link for individual‑level screening completion or an identity/logit link for site‑period Reach. An exchangeable working correlation will be specified, and inference will rely on site‑clustered robust standard errors with an appropriate small‑sample correction (e.g., the wild cluster bootstrap). Results will be presented as marginal effects with 95% confidence intervals. This approach provides valid inference under repeated cross‑sectional sampling and is well suited to the limited number of clusters in the study. As a sensitivity analysis, a generalized linear mixed model (GLMM) with a site‑level random intercept will be fitted to estimate the DiD effect. For binary outcomes, small‑sample inference will be obtained using bootstrap procedures, while for continuous outcomes the Kenward–Roger correction will be applied. These additional models will assess the robustness of findings to alternative correlation structures and modelling assumptions.

**Assumption checks:** The DiD analysis relies on the assumption that, in the absence of the intervention, intervention and control sites would have followed parallel trends. Because multiple pre‑intervention time points are not available, this assumption will be evaluated indirectly through baseline equivalence. Key sociodemographic and clinical characteristics will be compared between intervention and control sites using standardized mean differences, with values <0.10 indicating acceptable balance. Sensitivity analyses adjusting for any observed imbalances will be conducted, and additional robustness checks including alternative model specifications and negative‑outcome/placebo tests where feasible, will be used to assess the stability of the estimated intervention effect.

## 5.5 Secondary Analyses

Secondary analyses will address additional outcomes beyond the primary DiD estimate. Continuous outcomes, such as systolic blood pressure or site‑level control rates, will be analyzed using linear mixed‑effects models with appropriate adjustment for clustering. For count outcomes, including the number of completed follow‑ups or referrals, negative binomial mixed models will be used when overdispersion is detected. Effect modification will be explored by including interactions between the DiD term and key covariates such as sex, age group, primary language, and region of birth; where significant, stratified marginal effects will be reported. Implementation outcomes (AIM, IAM, and FIM scores) will be compared across sites using linear mixed models with site random effects; internal consistency will be examined using Cronbach’s α, and construct validity assessed through known‑groups comparisons (e.g., contrasting high‑fidelity versus low‑fidelity sites). Finally, to examine the relationship between implementation fidelity and effectiveness, multilevel models will be used to regress the primary effectiveness outcome on fidelity scores, adjusting for relevant covariates and site‑level clustering.

## 5.6 Missing Data Handling

**Primary approach:** Missing data will be addressed using multiple imputations by chained equations under the assumption that data are missing at random, including all outcomes, covariates, and site indicators.

**Sensitivity analyses:** Complete-case analyses and best-worst case scenario analyses will be performed for key outcomes to assess robustness of findings.

## 5.7 Multiplicity and Inference

- **Primary outcome:** Tested at α = 0.05 (two-sided).
- **Secondary outcomes:** Type I error will be controlled using the Benjamini–Hochberg false discovery rate procedure with q = 0.10.
- **Reporting:** Effect sizes will be reported with 95% confidence intervals and exact p-values.

## 5.8 Cost and Cost-Effectiveness Analysis

- **Perspective:** The economic evaluation will be conducted from the provider/health‑system perspective over the duration of the implementation period.
- **Resource measurement:** Resource use will be measured using micro‑costing methods combined with time‑driven activity‑based costing (TDABC) to capture labour, equipment, consumables, and workflow‑related time requirements.
- **Outcomes:** The primary economic outcomes will be the incremental cost per additional person screened and the incremental cost per additional person initiating treatment (ICER).
- **Uncertainty analysis:** Nonparametric bootstrap with 1,000 replicates; cost-effectiveness acceptability curves will be generated to assess probability of cost-effectiveness across willingness-to-pay thresholds.

# Section 6. Qualitative Integration and Mixed-Methods

**Mixed-Methods Analysis:** A convergent parallel mixed-methods design will be used, in which quantitative and qualitative analyses are conducted independently and integrated at the interpretation stage. This approach allows for complementary insights, with qualitative data providing context and explanation for quantitative findings.

**Integration Approach:** Quantitative and qualitative data will be integrated using joint displays, aligning quantitative outcomes with qualitative themes within each RE-AIM domain. For example, low follow-up rates may be contextualized with participant-reported barriers such as transportation challenges or limited appointment availability.

**Joint Interpretation:** Integrated findings will inform interpretation of effectiveness, adoption, fidelity, and sustainability outcomes. This approach facilitates identification of barriers and facilitators that may not be evident from quantitative data alone and helps explain variation in implementation success across sites.

**Iterative Feedback:** Insights from integrated analyses will be shared iteratively with sites to guide sustainment planning and support contextual adaptations of the intervention as needed.

**Documentation and Transparency:** All integration decisions, coding frameworks, and joint display outputs will be documented in an audit trail to ensure transparency, reproducibility, and accountability in reporting.

# Section 7. Data Governance, Ethics, And Cultural Safety

**Ethics approval**: University of Queensland human research ethics committee approval will be secured. Site permissions and informed consent with multilingual materials will be obtained from participating sites and participants.

**Data Governance and Security:**

- **De-identification:** Participant data will be de-identified at the source. Linkage keys will be stored separately and securely.
- **Access control:** Role-based access will limit data handling to authorized personnel only.
- **Encryption:** All electronic datasets will be encrypted during storage and transfer.
- **Data management:** Standard operating procedures will be followed for data entry, quality assurance, and secure storage. Audit trails will track data handling and any modifications.
- **Data sharing:** De-identified aggregate data and codebooks may be shared under formal data-sharing agreements. Qualitative data will be shared as summarized themes to preserve participant confidentiality.

**Cultural Safety:**

- Certified interpreters will be available for participants as required.
- Consent forms, surveys, and informational materials will be provided in relevant languages.
- Faith leaders and community advisors will be actively engaged in study planning, recruitment, and interpretation of findings.
- Study activities will be respectful of cultural practices regarding venues, gender, scheduling, and communication. Feedback loops will be established to address community concerns promptly.
