# Supplementary material for: Community-based model of care to improve access to and utilization of health services for common non-communicable diseases among culturally and linguistically diverse populations in Australia: an implementation research protocol
Source: Arch Public Health. 2026 Jun 10;84:186. doi: 10.1186/s13690-026-01986-0 (PMC13479535; doi:10.1186/s13690-026-01986-0)
Supplement: Supplementary file 2 [file 13690_2026_1986_MOESM2_ESM.docx]

**Community-based model of care to improve access to and utilization of health services for common non-communicable diseases among culturally and linguistically diverse populations in Australia: An implementation research protocol**

Ayelign Mengesha Kassie^1,2^, Aklilu Endalamaw^1,3^, Resham B Khatri^1^, Gregory Fowler^1^, Yibeltal Assefa^1^

**Author Affiliations:**

^1^School of Public Health, Faculty of Health, Medicine and Behavioral Science, The University of Queensland, Brisbane, Australia

^2^School of Nursing, College of Health Sciences, Woldia University, Woldia, Ethiopia

^3^College of Medicine and Health Sciences, Bahir Dar University, Bahir Dar, Ethiopia

**Corresponding author:** Ayelign Mengesha Kassie, Email: [ayelignmengesha59@gmail.com](mailto:ayelignmengesha59@gmail.com)

**Abstract**

**Background**: Australia's increasingly diverse population includes a nearly one third from Culturally and Linguistically Diverse (CALD) backgrounds, who often face significant challenges in accessing and utilizing healthcare services, particularly for non-communicable diseases (NCDs). These challenges stem from various factors including understanding and communication barriers in English, cultural differences, limited health literacy and healthcare system related limitations. This project aims to co-develop and test a culturally responsive community-based, multi-packaged model of care to enhance access to and utilization of health services for common NCDs (screening of hypertension and diabetes mellitus, and initiation of treatment for those diagnosed) among CALD communities in Australia.

**Methods**: This project will follow a four-phased approach, based on the Exploration, Preparation, Implementation, and Sustainment (EPIS) framework. Other complementary frameworks including the consolidated framework for implementation research will also be utilized depending on contextual requirements. In Phase I, formative research will be conducted through a scoping review, quantitative survey, and qualitative interviews with service users and healthcare providers to identify successful care models, service preferences, and key facilitators and/or barriers to service access and utilization. Phase II of the project will focus on co-developing a culturally responsive care model informed by the findings of the first phase, which will then be reviewed and standardized with input from stakeholders, including healthcare providers and CALD community members. The Phase III activities will involve capacity building and collecting baseline data and implementing the care model. Finally, in Phase IV, implementation outcomes will be assessed by collecting follow‑up data using the same tools from Phase III, but not necessarily from the same individuals. The quantitative follow up window for outcome assessment is 6 to 12 months after site activation, with any longer activity focused on qualitative work, sustainment assessments, and dissemination.

**Conclusions**: Overall, this project may play a pivotal role in enhancing the accessibility, quality, and cultural responsiveness of healthcare for CALD communities and reducing health disparities. We believe the lessons learned and the model developed through this process will be useful not only in Australia but also in other countries with similar multicultural communities.

**Keywords**: Culturally And Linguistically Diverse Communities; Community-Based Care Model; Implementation Research; Non-Communicable Diseases; Protocol

# **Contributions to the Literature**

- There is limited evidence on how culturally responsive, community-based models of care for non-communicable diseases can be developed and tested for CALD populations.
- This protocol presents a four-phased, adaptable implementation research protocol combining a scoping review, quantitative survey, and qualitative interviews to develop a culturally responsive care model.
- It shows how such models can be implemented and evaluated in real-world settings, with a focus on feasibility, acceptability, and health outcomes, and offers a practical framework that can be adapted to reduce inequities in chronic disease care in other contexts.

# **Background**

Mental illness, diabetes, and cardiovascular diseases are among the most prevalent chronic non-communicable diseases (NCDs) in Australia. Among these, mental and behavioural conditions were the most commonly reported in 2022, affecting 26.1% of the population (1). Hypertension is the leading risk factor for mortality in the country (2), and approximately one in three Australian adults has elevated blood pressure (2, 3). However, the condition remains underdiagnosed, and only about half of adults living with the disease are aware of their diagnosis (4, 5). Diabetes mellitus also affects around one in fifteen adults (6.6%) nationwide (6). Some reports indicate that the prevalence of such chronic diseases is higher among Culturally and Linguistically Diverse (CALD) communities compared to the general Australian population, particularly for hypertension (7), and type 2 diabetes mellitus with poor outcomes (8).

Overseas-born CALD populations are generally considered to be relatively healthy during their initial of years arrival in Australia, partly due to pre-migration health screening and other protective factors (9). However, the 2021 Australian Institute of Health and Welfare report on chronic health conditions has indicated that the prevalence of diseases of the heart, stroke, diabetes and kidney diseases were higher among CALD communities coming from several other countries than the Australian-born population. For instance, Bangladesh-born Australians are reported to have the highest prevalence of both diabetes and heart disease (12% and 4.6%, respectively) and kidney disease is highest in people born in Polynesian countries such as Tonga (1.9%) and Samoa (1.5%) (10). The burden of such chronic diseases has also been reported to be higher among people from CALD backgrounds as they age (9, 10).

The Australian healthcare system, though advanced, has not always been fully responsive to the needs of CALD communities (11). People from these communities often encounter difficulties in navigating health services due to language barriers, cultural misunderstandings, and limited health literacy (12, 13). As a result, CALD populations are less likely to seek timely medical care and may delay or even avoid health services due to the different challenges. For example, women from CALD backgrounds are less likely to have Cervical Screening Tests than people from the general population (14). Additionally, in Victoria, CALD patients were 51% less likely to utilize emergency medical services than non-CALD patients. Significant variations were also observed across age groups, countries of birth, and clinical presentations among these CALD service users (15). Another study has also indicated that emergency service use for acute coronary syndrome is lower among CALD than non-CALD populations in the region (61.9% vs 65.3%) (16).

The preferences of CALD communities regarding health services and delivery models might also differ from that of the general population in Australia due to differences in cultural backgrounds, languages, and prior healthcare experiences (17). This could be a key contributing factor for the existing disparities in health service utilization between CALD populations and the general Australian population, despite the ongoing efforts to improve access to health services for these communities (17, 18). As such, people from these communities are recognized as a priority population in several Australian Government policies (12). Therefore, understanding the patterns of disease and access to healthcare services within this diverse population is essential to effectively address their healthcare needs (12, 19).

Culturally and linguistically diverse communities possess unique facilitators such as strong social networks and community cohesion that can support greater engagement with health services and foster improved health outcomes (20). Thus, more culturally competent healthcare systems with more inclusive and effective care models tailored to meet the unique needs of Australia’s CALD communities are needed (21). Developing effective, culturally responsive models of care requires a thorough understanding of the specific preferences, barriers, and facilitators that influence these communities' healthcare experiences. Therefore, this project aims to co-develop and test a culturally responsive community-based model of care that can enhance access to and utilization of health services for common NCDs (screening of hypertension and diabetes mellitus, and initiation of treatment for those diagnosed) among CALD communities in Australia. Initially, we considered a broader focus on common NCDs, including cancers (bowel, cervical, skin, and breast), diabetes, hypertension, and common mental health conditions. However, the care pathways for these conditions differ significantly making it challenging to develop a single model of care and to assess implementation outcomes uniformly. Consequently, the study focus has been narrowed to diabetes and hypertension, as the lessons learned from such models can provide practical insights applicable to other NCD care more broadly.

The specific objectives are:

1) To identify the key facilitators and barriers that affect access to hypertension and diabetes mellitus screening services among CALD communities in Brisbane city, Australia.
2) To explore the preferences of CALD communities regarding health service delivery models that can enhance uptake of hypertension and diabetes mellitus screening services in Brisbane city, Australia.
3) To co-develop a culturally responsive community-based, multi-packaged, model of care that can help improve access to and utilization of health services for hypertension and diabetes mellitus screening, and initiation of treatment for those diagnosed among CALD communities in Brisbane city, Australia.
4) To implement and evaluate the implementation process (Reach, Effectiveness Adoption, Acceptability, Appropriateness, Cost, Feasibility, Fidelity, and Sustainability) and other outcomes of the multi-packaged care model.

**Research Questions**

A. What are the primary facilitators and barriers that affect access to hypertension and diabetes mellitus screening services among CALD communities in Brisbane city, Australia?
B. What are the preferences of CALD communities regarding health service delivery models that can enhance the uptake of hypertension and diabetes mellitus screening services in Brisbane city, Australia.
C. What elements should be considered in co-developing a culturally responsive community-based, multi-packaged, model of care to improve access to and utilization of health services for hypertension and diabetes mellitus screening, and initiation of treatment for those diagnosed among CALD communities in Brisbane city, Australia?
D. How can we implement the newly co-developed multi-packaged community-based model of care to enhance access to and utilization of health services for hypertension and diabetes mellitus screening, and initiation of treatment for those diagnosed among CALD communities in Brisbane city, Australia?
E. How effective and feasible is the multi-packaged model of care in improving access to and utilization of health services for hypertension and diabetes screening and initiation of treatment for those diagnosed among CALD communities, and what are its acceptability, cost, reach, sustainability, and other health outcomes?

# **Methods**

## **Study design and process**

This project will employ a four-phased strategy which has been utilized by other implementation research scholars with each phase focussing on distinct research objectives (22). A two‑period, before‑and‑after Difference‑in‑Differences design with repeated cross‑sectional sampling at baseline and follow‑up will be utilized to evaluate the impact of the implemented model of care on access to and utilization of hypertension and diabetes screening among CALD communities in Brisbane city. Australia. The Difference-in-Differences approach is a quasi-experimental method that can be utilized to estimate causal effects using observational data when randomization is not feasible. It helps compare the changes in outcomes over time between a treatment group (affected by the intervention) and a control group (not affected) (23-25). This design is appropriate for our study as it enables comparison of group‑level changes without requiring follow‑up of the same individuals over time. The Exploration, Preparation, Implementation, Sustainment (EPIS) framework will be utilized to guide the phase-wise execution of the overall implementation process of the project work. This framework provides a structured approach to understanding and navigating the complexities of implementing evidence based practices by considering multiple contextual factors (26). Additional complementary frameworks will also be utilized in different phases depending on the objectives of each phase and other contextual requirements.

***Phase I: Formative research***

We will conduct a scoping review in this phase. The scoping review will be helpful to identify successful care models and their implementation mechanisms in enhancing access to and utilization of health services for hypertension and diabetes screening and initiation of treatment for those diagnosed, from international contexts. For this objective, we will explore databases, including PubMed, Embase, Scopus, and Web of Science, to gather peer-reviewed papers. Additionally, other websites, and government reports will also be explored for grey literatures. Then, selected articles will be descriptively synthesized to highlight the attributes of successful community-based care models in enhancing access to and utilization of services for hypertension and diabetes mellitus screening. The overall process of the scoping review will be guided by the Preferred Reporting Items for Systematic reviews and Meta-Analyses extension for Scoping Reviews (PRISMA-ScR) Checklist (27). The findings of the literature review will be helpful in guiding the co-development of a culturally responsive community-based care model to improve access to and utilization of health services for hypertension and diabetes mellitus screening, and initiation of treatment for those diagnosed among CALD communities in Brisbane city, Australia.

In addition to the scoping review, a quantitative survey and in-depth interview with key informants will be conducted to address the first two specific objectives. The survey will be developed using Qualtrics, an online tool that enables collaborative survey design and response sharing. It will be used to assess the preferences of CALD communities for NCD health service delivery, and to identify the main barriers and facilitators to service utilization. Assuming a population of more than 10,000, a minimum sample size of 384 participants will be considered to ensure statistically meaningful results with a 95% confidence level and a 5% margin of error. For the qualitative component, we plan to conduct semi-structured interviews. The interview guide will be developed during Phase I based on findings from the scoping review and relevant literature. In addition, patient and public involvement (PPI) contributors will be engaged during the study to provide input and help ensure cultural and linguistic appropriateness. Our approach will be underpinned by a constructivist qualitative framework combined with experience-based co-design principles. This provides a theoretical foundation for exploring meaning-making, supporting co-production, and ensuring that community voices directly support the development of the care model.

Sampling for the qualitative interviews will follow a purposive strategy, aiming to capture diversity across ethnicity, language background, age, gender, and healthcare experience. We intend to recruit 12 to15 CALD service users and 12 to 15 healthcare providers using purposive sampling with the final numbers to be determined based on data richness and adequacy, rather than solely by the numerical targets. Participants will be recruited through the networking links of multicultural organisations, community networks, healthcare services, and community outreach across Brisbane with guidance from PPI contributors to ensure cultural appropriateness and effective engagement. A reflexive thematic analysis approach will be employed for the qualitative study, involving systematic coding, iterative theme development, and refinement to capture both shared experiences and culturally specific perspectives. This sequential process ensures that the co-development phase of the model of care is informed and guided by the findings of the quantitative survey, qualitative interviews, scoping review, and other literature collectively.

**Expected Outcomes of Phase I:** The expected outcomes of Phase I will be 1) identification of successful care models and their implementation mechanisms that can enhance access to and utilization of health services for hypertension and diabetes mellitus screening, and initiation of treatment for those diagnosed, 2) quantifying the key facilitators and barriers that enhance or hinder access to and utilization of health services for the diseases of interest among CALD communities in Brisbane city, Australia and 3) and exploring the preferences of these communities for health service delivery. These findings will be helpful in the development of the multi-packaged care model during Phase II. As such, the exploration component of the EPIS framework will be utilized to guide evidence synthesis in this formative phase research process. The process of exploration involves conducting a needs assessment, engaging stakeholders to gather diverse perspectives, reviewing existing evidence-based practices, and evaluating their feasibility within different organizational contexts (26) (**Fig 1**).

**The EPIS Framework**

**Figure** 1: The EPIS Farmwork. Source; EPIS Framework: What is EPIS? Retrieved from <https://episframework.com/what-is-epis> (28).

As shown in Fig 1, the EPIS Framework consists of four interrelated phases that guide the implementation of evidence-based practices (EBPs). In the Exploration phase, stakeholders assess emerging or existing health needs and determine which EBPs are best suited to address them, while also considering potential adaptations at various levels. The Preparation phase involves identifying barriers and facilitators within both the outer (e.g., policy, funding) and inner (e.g., organizational culture, leadership) contexts, planning adaptations, and designing implementation supports including coaching and feedback mechanisms. During the Implementation phase, the EBP is introduced into practice with support structures in place, alongside ongoing monitoring to ensure fidelity and allow for responsive adjustments. Finally, the Sustainment phase ensures that the necessary organizational and system supports remain active so the EBP continues to be delivered effectively over time, potentially with adaptation, to achieve lasting public health impact (28).

**Phase II:** Co-developing a culturally responsive, community-based care model for screening and initiating treatment for hypertension and diabetes, guided by CALD communities' preference to healthcare access in Australia. In this phase, the multi-packaged community-based care model will be co-developed in consultation with key stakeholders from CALD community members, religious or key community leaders, Brisbane city health department and other relevant bodies, based on the findings of Phase I, including the scoping review. Interventions that are identified as highly relevant, feasible and impactful from Phase I findings will be included in the preliminary model of care. Then, the preliminary model of care will be reviewed by 12 to 15 CALD service users, religious and other influential community leaders and 12 to 15 healthcare providers, managers and/or health department staffs to ensure the relevance and responsiveness of the care model.

Consequently, a series of workshops will be held with relevant stakeholders to review the included interventions, address potential challenges, and finalize the components of the multi-packaged model of care. Specifically, we will run three workshops: the first will involve the research team presenting key findings for discussion and prioritisation; the second will address co-development of potential components of the care model; and the third will focus on refining and validating the proposed model with participants. The developed multi-packaged model of care will cover various activities including behavioural, structural and system level interventions which will be implemented in Phase III. These interventions will then be first listed, mapped, and all the stakeholders need to reach on consensus on the components to be included on the final multi-packed model of care. Once the multi-packaged model of care is finalized, a review will be done by project advisory group. These will involve senior researchers and experts in the implementation research and project implementers from different sites.

This phase will be guided by the preparation phase component of EPIS framework. The intervention packages will also be standardised, and a monitoring strategy will be developed for the packages of care, once the model is developed. Standardisation will involve specifying the type and extent of standardisation required for each component. The scope and nature of this standardisation will be determined in collaboration with the project advisory group and various implementing stakeholders.

- The type of standardization for each component and step will be categorised as “mandatory”, “optional”, or “prohibited”. The “mandatory” section must be performed under all circumstances and, if not performed, will be considered as protocol deviation; the opposite is true for “prohibited”. On the other hand, the “optional” step may or may not be performed depending on the circumstances.
- In implementing the co-developed community-based care model, the standardization of each intervention component will be categorized as “exactly,” “with boundaries,” or “flexible (without boundaries),” based on the level of discretion allowed to healthcare providers. Components labelled “exactly” must be implemented without deviation. For example, the mandatory use of a standardized NCD screening checklists for hypertension and diabetes mellitus risk factors. Components categorized as “with boundaries” permit limited flexibility within predefined parameters, such as allowing follow-up intervals for hypertension suspected patients’ blood pressure re-check to range between 2 to 4 weeks based on clinical judgment. “Flexible” components allow full adaptation to local context, such as the choice of patient education methods, which may include group sessions, one-on-one counselling, or digital tools. This approach ensures a balance between consistency and contextual relevance, facilitating effective and adaptable service delivery.
- Intervention monitoring: The levels of implementation fidelity will be defined and framed in consultation with all the implementing stakeholders/sites. Implementation fidelity refers to the degree to which a programme or an intervention is delivered as is intended by project implementers (29). Therefore, understanding and measuring whether an intervention has been implemented with fidelity helps project implementers, practitioners and decision-makers gain a better understanding of how and why an intervention works, and the extent to which the health outcomes can be improved. Accordingly, any discrepancy in fidelity among implementing stakeholders will be resolved after discussion with each of them and arriving at a final consensus. In addition, a checklist and standard data collection form will be developed based on the multi-packaged interventions of the model of care. The checklist developed will be utilized to assess the level of implementation fidelity and compliance with the multi-packaged interventions employed to enhance access to and utilization of health services for hypertension and diabetes mellitus screening, and initiation of treatment for those diagnosed among CALD communities in Brisbane city, Australia.

**Expected outcomes of Phase II:** Co-development of a culturally responsive, community based, multi-packaged model of care to enhance access to and utilization of health services for hypertension and diabetes mellitus screening, and initiation of treatment for those diagnosed among CALD communities in Brisbane city, Australia. For instance, making services periodically available and accessible around faith-based settings, doing health promotions through community leaders and health professional facilitators, registry of target population and provision of services can potentially enhance access to and utilization of health services for hypertension and diabetes mellitus screening, and initiation of treatment for those diagnosed among CALD communities. Previous research in this area has demonstrated success in early detection and treatment for such disorders (30-32).

**Phase III:** Implementation of the community-based model of care. In this phase, the multi-packaged model of care co-developed in Phase II will be put in to action to enhance access to and utilization of health services for hypertension and diabetes mellitus screening, and initiation of treatment for those diagnosed among CALD communities in Brisbane city, Australia. This phase will be guided by the preparation and implementation components of EPIS Framework and the consolidated framework for implementation research (CFIR). The preparation component of EPIS will be utilized to guide the process of getting readiness for implementation. This includes developing training manuals and implementation evaluation checklists, building staff capacity through training, collaborating with implementing sites in developing infrastructure to support the practice, and carefully planning the implementation of the care model to fit the local context while maintaining fidelity. In addition, putting clear mechanisms to proactively identify potential barriers and strategize ways to address them will be part of preparation to ensure that the implementing organizations are well-positioned for successful execution. Baseline data collection will also be conducted at this time as part of the quasi-experimental study of the implementation process pilot testing.

Once the pre-implementation preparation phase is completed, the actual model implementation phase will be started in the selected faith-based community settings in collaboration with different health care providers in the city. These collaborating health facilities will be tasked to work with the faith-based CALD community leaders on ways of enhancing access to and utilization of health services for hypertension and diabetes mellitus screening, and initiation of treatment for those diagnosed. For the pilot testing purpose, the implementation process will focus on involving adults aged 40 years and above. Accordingly, we will be communicating with those religious organizations for their consent and cooperation once the ethical approval process is finalized and permissions are obtained from other concerned bodies in the health system. Then, people from these communities who were born overseas and are 40 years or older will be registered and invited for the service in collaboration with the project implementing health facilities. Additional key activities in this phase includes executing the plan, monitoring fidelity and outcomes through data collection, and using feedback for continuous improvement. Essential support systems such as coaching, supervision, and resource provision will be facilitated by the project advisory group in collaboration with the implementing site managers/coordinators during this phase, to sustain momentum and address challenges. Flexibility and responsiveness to real-time feedback will be emphasized to ensure that the multi-packaged interventions are delivered effectively, and adjustments are made when needed.

Planned activities that will be implemented in Phase III:

1) **Development of a structured training curriculum and training manual.** The project implementers will develop structured training modules based on the intervention packages requirements developed in Phase II and in consultation with other stakeholders. The training manuals will be contextualized to the practice change needs based on the preliminary findings and the co-developed multi-packaged model of care. The training sessions will be conducted using interactive sessions and practical demonstrations. A pre- and post-training assessment will be provided to the trainees to assess the practice and knowledge changes acquired from the training modules and identify the need for re-training before initiation of the project implementation process.
2) **Capacity building for project implementers:** The project steering committee (comprising of a multidisciplinary team of project implementers from different implementing sites) will provide a comprehensive training to the relevant stakeholders including the health workers who are directly involved in providing health care services for the selected NCDs. If this is not possible, the training will be provided by the project advisory members using online training platforms. The training will be provided with a combination of four to five interactive sessions, and didactic lectures to enhance the adoption of the multi-packaged interventions. The healthcare professionals and other stakeholders undergoing training will be assessed for knowledge and practice change using a pre-developed assessment module. A score of > 80% in the training assessment will be considered adequate and, if the score is < 80%, a re-training session will be provided (22).
3) **Implementation of the multi-packaged model of care:** Baseline data will be collected, and the model of care will be implemented in those settings in collaboration with different stakeholders, after completion of the trainings. The process of model implementation to enhance access to and utilization of health care services for hypertension and diabetes mellitus screening, and initiation of treatment for those diagnosed among CALD communities and other secondary objectives will be recorded. In addition, any challenges experienced in the adoption of the interventions will be documented and communicated for concerned bodies including the project implementing agencies.
4) **Identification of model implementation co-ordinators and facilitators:** We will be selecting one or more model implementation coordinators to run the overall implementation process. These individuals will be selected based on their experience and leadership qualities to promote good governance, accountability, and high-quality services. In addition, one facilitator will be selected at each implementing site. These facilitators will be drawn from experienced clinical leaders, health managers, and/or frontline staff members. These facilitators will be tasked to ensure the adoption of the multi-packaged interventions of the model of care being implemented in their respective settings. In addition, they will play a key role in guiding the model implementation process, monitoring progress, ensuring the quality and consistency of service delivery, facilitate timely data collection, and lead regular performance reviews. They will also assess whether interim or re-training is necessary at each site. Re-training will be scheduled if non-compliance with the protocols of the multi-packaged interventions is noted at a particular site as assessed by the implementation protocol checklist. If non-compliance is persistent, then it will be communicated to the project advisory group. They will also support in problem-solving at the facility level, encourage the use of evidence for decision-making, and ensure that reporting mechanisms are transparent and actionable. The project implementation advisory group will take the responsibility to identify these facilitators for each setting in collaboration with the site project implementing agencies. Subsequently, the project advisory group will conduct a process evaluation, and in-depth interviews will be carried out to explore the issues and challenges encountered in the implementation of these interventions, as described below.

**Process evaluation:** Process evaluation will be guided by the implementation component of EPIS framework and the CFIR. The CFIR has five constructs: intervention characteristics, outer setting, inner setting, characteristics of individuals, and process. These constructs serve as a foundation to explore the factors affecting implementation process, including barriers and facilitators of service delivery (33). Accordingly, the project advisory group will evaluate the compliance of implementing agencies and sites with the multi-component interventions included in the model of care. Participating sites will also assess whether the required structural and system changes have been implemented and whether healthcare professionals and other implementing staff have adopted the intended behavioural changes. These evaluations will be guided by the frameworks described above. Compliance monitoring at each site will be conducted by selected staff using a structured checklist, with results reported to the project advisory group on a quarterly basis.

If significant non-compliance with the implementation protocols is observed, a re-assessment will be conducted to evaluate the fidelity and acceptance of the model of care. Accordingly, in-depth interviews will be conducted with the health care professionals and other implementing agencies to better understand the issues they faced, and the challenges experienced during implementation of the multi-packaged interventions. These results of the findings will be utilized to make contextual adjustments on the provided list of interventions in the model of care or provide additional capacity building trainings. As such, the multi-packaged interventions will be either adapted as per the challenges noted or co-developed as per the site needs. Otherwise, retraining will be provided if retraining alone is sufficient. Then, a final assessment will be conducted to assess the implementation process and clinical outcomes of implementing the multi-packaged model of care in enhancing access to and utilization of health services for hypertension and diabetes mellitus screening and initiation of treatment among CALD communities (34) (**Table 1**).

**Table 1:** Model co-development, preparation and implementation considerations with proposed implementation outcomes, from complementary frameworks perspective.

**Assessment requirements of domains for model implementation: CFIR perspective**

**Multi-packaged components of the care model**

**Strategies to address identified gaps**

**Model Implementation Outcomes**

**Intervention Characteristics**

**Inner setting**

**Outer setting**

**Individual characteristics**

**Process**

Intervention source, evidence strength and quality, relative advantage, adaptability, trialability, complexity, design quality and packaging, and cost.

Patient needs and resources, cosmopolitanism, peer pressure, and external policy and incentives.

Structural characteristics, networks and communications, culture, implementation climate, and readiness for implementation.

knowledge and beliefs about the intervention, self-efficacy, individual stage of change, individual identification with organization, and other personal attributes (i.e., skills and knowledge, motivation, etc.).

Planning, engaging, executing, and reflecting and evaluating.

Reach

Effectiveness

Adoption

Implementation:

- Acceptability

- Appropriateness

- Feasibility

- Cost

- Fidelity

Satisfaction with service

Mainly RE-AIM framework perspective outcomes

Client experiences perspective outcome

**Community-based model of care**

Sustainability**:**

- Continuity of care

- Adherence

- Lost follow-up

**Phase IV:** Post-intervention phase. This phase will involve collecting data in order to evaluate the implementation outcomes of the multi-packaged model of care. The effectiveness of these complex interventions in their specific settings will be evaluated by the different stakeholders involved in the implementation process. This will include assessing the implementation process outcomes and impacts of the model of care in enhancing access to and utilization hypertension and/or diabetes screening services among CALD communities. For this reason, we will collect quantitative data to measure model implementation impacts in terms of access enhancement, utilization rate and other clinical outcomes. In addition, we will be conducting qualitative interviews with the different stakeholders and collaborators in order to evaluate their thoughts about the feasibility, sustainability and potential long-term impacts of the implemented multi-packaged model of care (**Fig 2**).

**Phase-I: Formative research**

**Phase-II: Development of a multi-packaged model of care**

**Phase-III: Implementation of the multi-packaged model of care and process evaluation**

**Phase-VI: Outcome assessment and dissemination**

**Research question:** How is the acceptability, appropriateness, fidelity feasibility and other impacts of the implemented multi-packaged model of care in enhancing access to and utilization of health services for those diseases among CALD communities?

**Research objective:** To assess the reach, effectiveness, acceptability, appropriateness, feasibility, fidelity, cost and other outcomes of the implemented care model.

**Activities:** Post-intervention data collection, analysis and dissemination.

**Research questions:** What are the attributes of successful community-based care models? What are the main barriers and facilitators that impact access to and utilization of health services and the preferences of CALD communities for health service delivery in Australia?

**Research objective:** To identify the attributes of successful community-based care models and identify the main the barriers and facilitators that impact access to and utilization of health services and the preferences of CALD communities for health service delivery in Australia?

**Activities**: Conduct scoping review, quantitative survey and qualitative interviews.

**Research question:** What elements should be incorporated in co-developing a culturally responsive community-based model of care to improve access to and utilization of health services for hypertension and diabetes mellitus screening, and initiation of treatment among CALD communities?

**Research objective:** To co-develop a mult-packaged, community-based, model of care that can improve access to and utilization of health services for those diseases.

**Activities:** Multi-packaged community-based care model co-development.

**Research question:** How can we implement the developed multi-packaged model of care to enhance access to and utilization of health services for those diseases among CALD communities?

**Research objective:** To implement the newly developed culturally responsive community-based model of care on CALD community members to improve access to and utilization of services.

**Activities:** Training module development, capacity building for implementers, actual implementation of care model, process evaluation etc.

**Figure 2.** Phase-wise research strategy, model co-development and implementation process to enhance access to and utilization of hypertension and/or diabetes screening services among CALD communities in Australia.

## **Outcomes of model implementation**

The primary model implementation impact outcome is “Reach”, or the proportion of eligible CALD adults completing hypertension and/or diabetes screening. The primary implementation process outcomes are acceptability, appropriateness, and feasibility of the model of care, measured at the site and stakeholder level. Secondary outcomes include follow-up completion, treatment initiation, clinical control (BP and glycemia), intervention fidelity, program cost, and sustainability. Baseline measurements will be collected prior to implementation, with follow-up assessments occurring at 6 to 12 months post-implementation depending on the specific outcome (**See Supplementary File 1**). The sustainment component of EPIS framework and the RE-AIM evaluation framework will be utilized to guide assessment of these implementation outcomes related with implementation of the multi-packaged model of care. The RE-AIM framework is a widely used planning and evaluation tool in public health that helps assess the impact and scalability of health interventions across five key dimensions: Reach (the extent to which the target population participates), Effectiveness (the impact on important outcomes), Adoption (the extent to which settings and staff adopt the intervention), Implementation (how well the intervention is delivered as intended), and Maintenance (the extent to which a program is sustained over time) (35). Originally developed to bridge the gap between research and real-world application, RE-AIM guides both the design and evaluation of programs to ensure they are not only effective in controlled trials but also practical and sustainable in everyday settings. However, some of the implementation outcomes in this study, specifically acceptability, appropriateness, feasibility, fidelity and cost are not native RE-AIM domains. These outcomes are intentionally drawn from Proctor et al.’s taxonomy to complement RE‑AIM’s implementation dimension with complementary measures being mapped pragmatically across the two frameworks (35, 36). Reach will be calculated as the number screened divided by the number invited within each site and period based on community registries and screening logs. In addition, individuals who screen positive during each measurement period will form a nested cohort for follow up, and these clinical pathway outcomes will be summarised and analysed as site period proportions to remain consistent with the repeated cross-sectional design.

**Proposed tools for measuring implementation outcomes**

There are various tools that have been utilized to assess various implementation outcomes across the literature. We will be using different adapted tools including the Acceptability of Intervention Measure (AIM), Intervention Appropriateness Measure (IAM), and Feasibility of Intervention Measure (FIM), psychometrically validated tools developed to assess key early-stage implementation outcomes. Each of these instruments consist of four items, rated on a five-point Likert scale from "completely disagree" to "completely agree." The AIM captures the degree to which stakeholders find an intervention agreeable or satisfactory, making it a useful measure of perceived acceptability. The IAM evaluates whether an intervention is perceived as a good fit or relevant for a given context or problem, thereby assessing its appropriateness. The FIM measures the perceived practicality and ease with which an intervention can be successfully implemented in a specific setting. These tools are designed for ease of use, brevity, and reliability, making them highly suitable for integration into implementation research and practice across diverse settings (37). In addition, other tools will be utilized depending on the expected outcome measures including the Clinical Sustainability Assessment Tool (CSAT), Short Form, and the Program Sustainability Assessment Tool (PSAT), Short Form, for model implementation sustainability assessment (**Table 2**).

**Table 2:** Implementation outcomes and proposed assessment tools for the model of care.

| RE‑AIM Implementation Evaluation Framework with Proctor et al. style implementation outcomes | Constructs | Assessment tools/Measures | Source of data | Method |
| --- | --- | --- | --- | --- |
|  | Reach | Participation rate: % of individuals invited who actually undergo screening for the target NCDs. | Registry lists and clinical records | - |
|  | Effectiveness | Follow-up rate: % of screen-positive individuals who receive timely confirmatory diagnosis and/or treatment. | Clinical records | - |
|  |  | Proportion of people with controlled hypertension and diabetes among those diagnosed. | Clinical records | - |
|  |  |  |  | - |
|  | Adoption | Proportion of implementing agents/staffs  that deliver model of care components. | Clinical records | - |
|  | Implementation |  |  |  |
|  | - Acceptability | Acceptability of Intervention Measure (AIM) 4-items (37). | Implementing site staff members | Survey |
|  | - Appropriateness | Intervention Appropriateness Measure (IAM) 4-items (37). | Implementing site staff members | Survey |
|  | - Feasibility | Feasibility of Intervention Measure (FIM) 4-items (37). | Implementing site staff members | Survey |
|  | - Cost | Cost capture survey (38), and time-driven activity-based costing methods (39). | Research team and implementing site staff members | Survey |
|  | - Fidelity | A checklist developed based on the interventions of the developed model of care package. | Implementing site staff members/leaders | Survey |
|  |  |  |  |  |
| EPIS and RE-AIM Framework | Sustainment | Clinical Sustainability Assessment Tool  (CSAT) Short Form: 7 domains, 21 items total (40). | Implementing sites | Survey |
|  |  | Program Sustainability Assessment Tool  (PSAT) Short Form: 8 domains, 40 items total (40). | Implementing sites | Survey |
|  |  | And/or other program-based tools. | Implementing sites | Survey |

## **Cluster size and number of expected participants**

We are proposing to include six faith-based organisations having large CALD community followers as intervention sites, as these settings provide trusted community environments that are well suited for delivering an outreach model of care. A matched set of six comparable faith-based organisations will be selected as control sites to allow for meaningful comparison. Faith-based organisations are institutions that are guided by religious principles and provide prayer and other religious activities, as well as services such as social support, education, and healthcare. However, while some of such organizations might focus on specific CALD communities, many may offer services to the wider population regardless of cultural or religious background. Therefore, we are aiming to consider such faith-based settings, and the final selection of which specific organisations to include and not to include will be decided during the later stages of model co-development process, based on relevance and feasibility. All selected organisations will be invited to participate and collaborate throughout the implementation research process, including planning and co-developing the intervention components. Comparability between intervention and control sites will be ensured by selecting organisations with similar CALD population size, demographic characteristics, and levels of community engagement, thereby supporting baseline equivalence across clusters and the assumptions required for a robust Difference-in-Differences approach-based data analysis. The details of the implementation process, including the selection of intervention and control sites, will be determined once the components of the multi-packaged model of care are identified and the model of care is co-developed in consultation with relevant stakeholders.

With regards to the sample size for this quasi-experimental study using the Difference-in-Differences approach, we have considered a hypertension prevalence of 34%, and an assumed baseline screening rate of 50% based on previous literature which shows that only one in two people detected to have hypertension are aware of their condition among the Australian population (4, 5). Our target is to increase hypertension screening rates among CALD communities from 50% at baseline to at least 70 % post-intervention, representing a 20% increase. To detect this difference with 80% power and a 5% significance level, we first calculated the sample size for comparing two independent proportions, assuming individual randomization. This yielded an unadjusted sample size of approximately 182 participants (91 per group).

Nevertheless, given the study’s clustered design involving 12 sites (six intervention and six control clusters), it is necessary to adjust for the expected intra-cluster correlation (ICC), which accounts for the similarity of responses within clusters. This adjustment is done by applying a design effect, calculated as: Design Effect (DE) = 1 + (m − 1) × ICC, where “m” is the average number of participants per cluster. In this study, we expect to recruit 100 participants per cluster and assume an ICC of 0.02 (41). Applying these values, the design effect becomes: DE = 1 + (100 − 1) × 0.02 = 1 + 99 × 0.02 = 2.98. Applying this yielded an adjusted total sample size of approximately 543 participants, or about 272 per arm. Therefore, with the 100 expected participants from each cluster, the final planned sample size of 1,200 exceeds the minimum requirement and ensures sufficient power to detect the expected effect while remaining practical. Furthermore, the qualitative component will also include in-depth interviews, and stakeholder workshops with CALD communities, healthcare providers, and policymakers to explore perceptions, implementation processes, barriers, and enablers.

## **Data analysis plan for the main model**

The Difference in Differences approach will be used to compare changes in hypertension and diabetes screening uptake between intervention and control sites over time with a repeated cross-sectional design. A Generalized Estimating Equations model will be utilized as the primary analysis method with site clustered robust standard errors and an appropriate small sample correction to account for the limited number of clusters. In addition, a generalized linear mixed model with a site level random intercept will be used as a sensitivity analysis. Key participant characteristics including age, sex, region of birth, education, primary language, and baseline comorbidities will be included as covariates. Results will be presented as adjusted marginal effects with ninety five percent confidence intervals. Baseline equivalence between intervention and control sites will be examined using standardized mean differences, and any identified imbalances will be controlled for in the modelling. The parallel trends assumption will be assessed indirectly through these baseline comparisons and sensitivity analyses as only one pre intervention time point is available. For secondary outcomes, follow up completion, treatment initiation, and clinical control will be derived from the nested cohort of screen positive individuals and will be summarised and analysed as site period proportions. Missing data will be handled using multiple imputation with additional sensitivity analyses to evaluate the robustness of findings. If individual level data are pooled across sites with fixed per site samples, design weights proportional to the site population of eligible invitees will be used to account for unequal selection probabilities (**See Supplementary File 1**).

# **Patient and public involvement**

No patients or members of the public were involved in developing this protocol. Nevertheless, PPIs, CALD community members, religious and community leaders, representatives from the Brisbane City health department, and other relevant bodies will be engaged during Phase I and subsequent stages to ensure that the research and the proposed model of care are culturally and contextually appropriate, based on their contextual requirements.

# **Work plan**

This implementation research is designed to be carried out over a five‑year period to ensure the systematic planning, phased execution, and rigorous evaluation of all project components. The quantitative follow up window for outcome assessment is 6 to 12 months after site activation, with any longer activity focused on qualitative work, sustainment assessments, and dissemination. A comprehensive description of the overarching operational plan, including methodological procedures, timelines, and implementation strategies, is provided in Supplementary File 1. In addition, the overall plan has been schematically depicted as follows to further aid conceptualization of the study design (**Fig 3**).

Post-intervention/ implementation outcome assessment phase: **1 year**

Implementing the co-developed model of care:

**1 and half years**

Pre-intervention phase: **1 and half years**

Co-development of a multi-packaged community-based care model:

**1 year**

**Figure 3:** Duration of the different phases in the implementation research process to enhance access to and utilization of health services for the target NCDs among CALD communities in Brisbane city, Australia.

# **Limitations**

A potential limitation of this protocol is its focus on faith-based settings, which may limit the generalisability of the co-developed model to secular or non-religious contexts and to CALD communities who do not engage with religious institutions. While some of these religious organisations (e.g., churches, mosques, and temples) may focus on specific CALD communities, many provide services to the wider population irrespective of cultural or religious background. Therefore, the selection of specific organisations in the final stages will depend on relevance and feasibility considerations, which may limit the representativeness of the settings included.

# **Abbreviations**

AIM: Acceptability of Intervention Measure; EBP: Evidence Based Practice; CALD: Culturally and Linguistically Diverse; CFIR: Consolidated Framework for Implementation Research; CSAT: Clinical Sustainability Assessment Tool; EPIS: Exploration, Preparation, Implementation, and Sustainment; FIM: Feasibility of Intervention Measure; ICC: Intra-Cluster Correlation; IAM: Intervention Appropriateness Measure; NCDs: Non-Communicable Diseases; PRISMA-ScR: Preferred Reporting Items for Systematic Reviews and Meta-Analyses Extension for Scoping Reviews; PSAT: Program Sustainability Assessment Tool; RE-AIM: Reach, Effectiveness, Adoption, Implementation, and Maintenance.

# **Declaration**

# **Ethical approval and consent to participate**

Ethical clearance will be first obtained from The University of Queensland Human Research Ethics committee. Then, additional approvals and/or permissions will be sought from collaborating health systems/departments. In addition, consent will be obtained from CALD community study participants, healthcare professionals and participating sites in implementing the multi-packaged model of care. Data collected from study participants will be kept confidential and will be recorded in anonymized form during and after the study. Electronic data will be stored in a password protected drive, and so only able to be accessed by the members of the research team. Furthermore, the results of this research project are expected to be published and/or presented in various forms. During this process, the information provided will be reported anonymously and in a non-identifiable manner.

**Consent for publication**: Not applicable

**Availability of data and materials**: Not applicable

**Competing interests**: The authors declare no competing interests.

**Funding**: Not Applicable

**Acknowledgment**

Not applicable

**Author contributions**

AM, AE and YA conceived the idea and designed the protocol. All other authors participated in reviewing the protocol. AM wrote the first draft of the manuscript. YA, AE, RK and GF, reviewed the first draft and provided valuable feedback. AM revised the first draft after receiving comments from the co-authors and all authors reviewed, edited, and approved the submission of the final protocol.

# **References**

1.	Australian Bureau of Statistics. Health conditions prevalence [Internet]. Canberra: ABS; 2022 [cited 2025 May 19]. Available from: <https://www.abs.gov.au/statistics/health/health-conditions-and-risks/health-conditions-prevalence/latest-release>.

2.	Australian Cardiovascular Alliance. Hypertension [Internet]. Melbourne: Australian Cardiovascular Alliance; [cited 2025 May 19]. Available from: <https://ozheart.org/strategic-initiatives/clinical-themes-initiative/hypertension/>.

3.	Healthdirect Australia. High blood pressure (hypertension) [Internet]. Canberra (AU): Healthdirect Australia; 2024 [cited 2025 May 19]. Available from: <https://www.healthdirect.gov.au/high-blood-pressure-hypertension>.

4.	The George Institute for Global Health. Shop-to-Stop Hypertension: A public screening campaign to detect and manage raised blood pressure in Australian adults. Available from: <https://www.georgeinstitute.org/our-research/research-projects/shop-to-stop-hypertension-a-public-screening-campaign-to-detect-and-manage-raised-blood-pressure-in>, (Accessed on May 26, 2025).

5.	Carnagarin R, Nolde JM, Yang J, Marques FZ, Picone DS, Lambert GW, et al. Stagnating rates of blood pressure control in Australia: insights from opportunistic screening of 10 046 participants of the May Measurement Month campaigns. Journal of Hypertension. 2023;41(4):632-7.

6.	Australian Bureau of Statistics. Rise in proportion of Australians with diabetes [Internet]. Canberra: ABS; 2025 March 31 [cited 2025 May 19]. Available from: <https://www.abs.gov.au/media-centre/media-releases/rise-proportion-australians-diabetes>.

7.	Queensland Health. Exploring the health of culturally and linguistically diverse (CALD) populations in Queensland: 2016–17 to 2019–20 [Internet]. Brisbane: Queensland Health; 2023 Mar [cited 2025 May 19]. Available from: <https://www.health.qld.gov.au/__data/assets/pdf_file/0028/1217593/QGV0439_CALD-Populations-Report_Full-Report.pd>.

8.	Tewari S, Lin SS. Managing diabetes in CALD communities [Internet]. Endocrinology Today. 2019 Mar;8(1):28–31 [cited 2025 May 19]. Available from: <https://endocrinology.medicinetoday.com.au/et/2019/march/regular-series/managing-diabetes-cald-communities>.

9.	Huang G, Guo F, Taksa L, Cheng Z, Tani M, Liu L, et al. Decomposing the differences in healthy life expectancy between migrants and natives: the ‘healthy migrant effect’ and its age variations in Australia. Journal of Population Research. 2023;41(1):3.

10.	Health AIo, Welfare. Chronic health conditions among culturally and linguistically diverse Australians, 2021. Canberra: AIHW; 2023.

11.	Henderson S, Kendall E. Culturally and linguistically diverse peoples’ knowledge of accessibility and utilisation of health services: exploring the need for improvement in health service delivery. Australian journal of primary health. 2011;17(2):195-201.

12.	Australian Institute of Health and Welfare. Culturally and linguistically diverse Australians-Overview. Available from: <https://www.aihw.gov.au/reports-data/population-groups/cald-australians/overview>, (Accessed on April 2, 2025).

13.	Gartoulla P, Joshi A. Key organisations for working with culturally and linguistically diverse families: Australian Institute of Family Studies. Available from: <https://aifs.gov.au/resources/resource-sheets/key-organisations-working-culturally-and-linguistically-diverse-families>, (Accessed on May 05, 2025).

14.	Australian Government Department of Health. People from culturally and linguistically diverse backgrounds. Available from: <https://www.health.gov.au/our-work/ncsp-healthcare-provider-toolkit/working-with-patients/culturally-and-linguistically-diverse-backgrounds>, (Accessed on April 24, 2025).

15.	Le PH, Cox S, Delir Haghighi P, Wybrow M, Smith K, Nehme Z. Utilization of emergency medical services by Culturally and Linguistically Diverse patients: a population-based retrospective study. Prehospital Emergency Care. 2024;28(8):988-97.

16.	Olani AB, Eastwood K, Howell S, Bray JE. Emergency Medical Service Use for Acute Coronary Syndrome in Culturally and Linguistically Diverse Immigrant Populations. Heart, Lung and Circulation. 2025.

17.	Javanparast S, Naqvi SKA, Mwanri L. Health service access and utilisation amongst culturally and linguistically diverse populations in regional South Australia: a qualitative study. Rural and Remote Health. 2020;20(4):1-15.

18.	Marcus K, Balasubramanian M, Short S, Sohn W. Culturally and linguistically diverse (CALD): terminology and standards in reducing healthcare inequalities. Australian and New Zealand Journal of Public Health. 2022;46(1):7-9.

19.	Wohler Y, Dantas JA. Barriers accessing mental health services among culturally and linguistically diverse (CALD) immigrant women in Australia: Policy implications. Journal of immigrant and minority health. 2017;19:697-701.

20.	Engaging Culturally and Linguistically Diverse Communities. A guide for the Western Australian Public Sector. Available from: <https://www.omi.wa.gov.au/resources-and-statistics/publications/publication/engaging-culturally-and-linguistically-diverse-communities>, (Accessed on June 27, 2025).

21.	Khatri RB, Assefa Y. Access to health services among culturally and linguistically diverse populations in the Australian universal health care system: issues and challenges. BMC Public Health. 2022;22(1):880.

22.	Rohilla R, Gupta M, Anish TS, Cherian JJ, Singh MP, Kakkar AK, et al. Multipronged interventions to reduce surgical site infections: A multicenter implementation research protocol. PloS one. 2025;20(3):e0319645.

23.	Dimick JB, Ryan AM. Methods for evaluating changes in health care policy: the difference-in-differences approach. Jama. 2014;312(22):2401-2.

24.	Saeed S, Moodie EE, Strumpf EC, Klein MB. Evaluating the impact of health policies: using a difference-in-differences approach. International journal of public health. 2019;64:637-42.

25.	Callaway B. Difference-in-differences for policy evaluation. Handbook of labor, human resources and population economics. 2023:1-61.

26.	Moullin JC, Dickson KS, Stadnick NA, Rabin B, Aarons GA. Systematic review of the exploration, preparation, implementation, sustainment (EPIS) framework. Implementation Science. 2019;14:1-16.

27.	McGowan J, Straus S, Moher D, Langlois EV, O’Brien KK, Horsley T, et al. Reporting scoping reviews-PRISMA ScR extension. J Clin Epidemiol. 2020;123(177):e9.

28.	EPIS Framework. (n.d.). What is EPIS? Available from <https://episframework.com/what-is-epis>, (Accessed on May 18, 2025).

29.	Carroll C, Patterson M, Wood S, Booth A, Rick J, Balain S. A conceptual framework for implementation fidelity. Implementation science. 2007;2:1-9.

30.	Pastakia SD, Ali SM, Kamano JH, Akwanalo CO, Ndege SK, Buckwalter VL, et al. Screening for diabetes and hypertension in a rural low income setting in western Kenya utilizing home-based and community-based strategies. Global Health. 2013;9:21.

31.	Al-Ghamdi AJ, Al-Turki KA, Al-Baghli NA, El-Zubaier AG. A community-based screening campaign for the detection of diabetes mellitus and hypertension in the eastern province, saudi arabia: methods and participation rates. J Family Community Med. 2007;14(3):91-7.

32.	Nonterah EA, Chatio ST, Willis A, Alale JA, Zakariah-Akoto S, Darko N, et al. Hypertension and diabetes control: faith-based centres offer a promise for expanding screening services and linkage to care in Ghana. BMC Primary Care. 2024;25(1):382.

33.	Rangachari P, Mushiana SS, Herbert K. A scoping review of applications of the Consolidated Framework for Implementation Research (CFIR) to telehealth service implementation initiatives. BMC Health Services Research. 2022;22(1):1450.

34.	Damschroder LJ, Reardon CM, Widerquist MAO, Lowery J. The updated Consolidated Framework for Implementation Research (CFIR): guidelines and tools for implementing complex innovations. Available from: <https://cfirguide.org/constructs/>, (Accessed on April 29, 2025).

35.	D'Lima D, Soukup T, Hull L. Evaluating the application of the RE-AIM planning and evaluation framework: an updated systematic review and exploration of pragmatic application. Frontiers in Public Health. 2022;9:755738.

36.	RE-AIM Workgroup. WHAT ARE RE-AIM AND PRISM? Available from: <https://re-aim.org/>, (Accessed on May 12, 2019).

37.	Weiner BJ, Lewis CC, Stanick C, Powell BJ, Dorsey CN, Clary AS, et al. Psychometric assessment of three newly developed implementation outcome measures. Implementation science. 2017;12:1-12.

38.	Ritzwoller DP, Sukhanova A, Gaglio B, Glasgow RE. Costing behavioral interventions: a practical guide to enhance translation. Annals of Behavioral Medicine. 2009;37(2):218-27.

39.	Kaplan RS, Witkowski M, Abbott M, Guzman AB, Higgins LD, Meara JG, et al. Using time-driven activity-based costing to identify value improvement opportunities in healthcare. Journal of Healthcare Management. 2014;59(6):399-412.

40.	Malone S, Prewitt K, McKay V, Zabotka L, Bacon C, Luke DA. Lowering the burden: Shorter versions of the Program Sustainability Assessment Tool (PSAT) and Clinical Sustainability Assessment Tool (CSAT). Implementation Science Communications. 2024;5(1):113.

41.	Lewis J, Julious SA. Sample sizes for cluster-randomised trials with continuous outcomes: accounting for uncertainty in a single intra-cluster correlation estimate. Statistical methods in medical research. 2021;30(11):2459-70.
